# The Immediate Post-Operative Radiograph is an Unreliable Measure of Coronal Plane Alignment in Total Knee Replacement

**DOI:** 10.3389/fsurg.2014.00035

**Published:** 2014-09-05

**Authors:** Joshua Petterwood, Michelle M. Dowsey, Daevyd Rodda, Peter F. M. Choong

**Affiliations:** ^1^Department of Orthopaedics, St. Vincent’s Hospital Melbourne, The University of Melbourne, Melbourne, VIC, Australia; ^2^Department of Surgery, St. Vincent’s Hospital Melbourne, The University of Melbourne, Melbourne, VIC, Australia

**Keywords:** knee, alignment, long-leg radiographs, knee replacement, mechanical axis

## Abstract

**Background:** Restoration of a neutral mechanical axis is a primary goal of total knee replacement (TKR). A mechanical axis within 3° of neutral has been correlated with improved implant longevity, function, and patient satisfaction. We hypothesize that the immediate post-operative radiograph is an unreliable method of measuring alignment following TKR surgery.

**Methods:** Seventy-five consecutive patients had supine X-rays performed on day two post-operatively followed by standing long-leg radiographs (LLRs) 6 weeks post-operatively. Correlation was sought between the mechanical axis measured on the LLR and surrogate markers of alignment on the post-operative X-ray including component alignment and an estimation of anatomical alignment using the available length of femoral and tibial shafts. Inter- and intra-observer reliabilities were assessed.

**Results:** The mean mechanical axis on the LLR was 180.5 (SD 3.0, range 175.1–187.1). Mean offset between anatomical axis and mechanical axis was 6.4°. The mean anatomical axis measured on the short-leg X-ray was 174.9 (SD 2.4, range 169.5–181.3). Mechanical axis on the LLR was compared to the anatomical axis measured on the short-leg radiograph (SLR) + 6° with an interclass correlation coefficient of 0.588 (*p* < 0.001). The level of disagreement between the short- and long-leg X-rays was assessed using the Bland–Altman method and demonstrated clinically important discrepancies of 5 or more degrees in 9% of cases. Inter- and intra-observer agreements were high on all measures (*p* < 0.001).

**Conclusion:** The long-leg weight bearing X-ray is an essential tool to accurately assess coronal plane alignment post TKR. While the immediate post-operative X-ray taken supine provides useful information to the surgeon on any immediate complications, our results indicate that it cannot be relied upon to determine correct restoration of the mechanical axis.

## Introduction

One of the primary aims of total knee replacement (TKR) surgery is to restore the overall mechanical axis of the lower limb. Restoration of the mechanical axis to within 3° of neutral has been shown to increase implant longevity, improve function, and lead to higher levels of patient satisfaction (1–4). Assessment of coronal plane alignment is best performed using the standing long-leg radiograph (LLR), which enables measurement of the mechanical axis through visualization of the hip, knee, and ankle. While some limitations exist the LLR remains the gold standard in the assessment of coronal plane alignment (5–8). Previous work from this institution has shown the LLR to be a more accurate measure of alignment than both CT scan and intra-operative computer navigation (5).

Despite the well-documented importance of restoring the mechanical axis, few surgeons use the LLR radiograph post-operatively to assess implant positioning and overall coronal plane alignment. Most often, surgeons rely upon the initial post-operative X-ray taken in hospital with the patient in a supine position to evaluate component positioning and estimate alignment. Multiple factors may contribute to inaccuracies in the assessment of alignment on the short-leg post-operative X-ray including the inability to achieve full extension, variation in posterior tibial slope, the inability to adequately achieve neutral rotation and, most importantly, the lack of weight bearing.

Assessment of coronal alignment in normal or osteoarthritic knees prior to knee replacement surgery has been performed on standard weight bearing X-rays of the knee alone. High correlation between the anatomical axis measured on the knee X-ray and the mechanical axis on the LLR has been shown by a number of authors (9, 10).

To our knowledge, a comparison of the initial post-operative X-ray and the LLR in assessing alignment in TKR has not previously been undertaken.

It is important that surgeons, outside of a research setting where the LLR is not routinely used, are able to accurately assess alignment following TKR surgery. We hypothesize that the initial post-operative X-ray cannot be used to assess alignment in TKR surgery and suggest that the LLR should become the standard of care for all patients.

## Materials and Methods

### Subjects

In the 6-month period, January 2009 to July 2009, 75 consecutive patients with advanced degenerative osteoarthritis underwent unilateral primary TKR. Two prostheses were used – the Scorpio (Stryker, Kalamazoo, MI, USA) and the PFC Sigma (DePuy Orthopedics, Warsaw, IN, USA) aiming for a neutral anatomical alignment. Conventional jigs (intra-medullary femur, extra-medullary tibia) were used in 73/75 cases with navigation used in two cases. Institutional ethics approval was obtained for this study.

### Radiographs

All patients had supine X-rays of the knee performed on day two post-operatively according to a standardized protocol. Following discharge, all patients underwent LLRs between 6 weeks and 3 months post-operatively.

The initial post-operative radiograph was taken supine with the knee in full extension ensuring that there was no internal or external rotation by referencing the malleoli. At day two, not all patients are able to fully extend the knee and all efforts were taken to ensure the maximal degree of extension possible with bandages removed. A 35 cm × 43 cm cassette was used to better visualize alignment of the leg rather than a 24 cm × 30 cm cassette used for standard knee X-rays. The radiographic beam was centered on the knee from a distance of approximately 100 cm with a setting of 4–8 mA s and kilovoltage of 60–65 kVp.

The LLR was taken using a custom Perspex stand designed and constructed to standardize LLRs. This stand ensured that the malleoli were 10 cm apart, and attempted to prevent outliers in rotation by grossly positioning the feet and knee, and hence the flexion axis of the knee in the sagittal plane, parallel with the X-ray beam. The radiograph was taken using three 430 mm × 36 mm cassettes with a graduated grid, with the limb fully extended. The radiographic beam was centered on the knee from a distance of approximately 270 cm with a setting of 32 mA s and kilovoltage of 77–95 kVp dependent on the patient’s limb size (11).

### Measurement of angles

All angles were measured on digital radiographs using the PACS system’s Cobb angle measure (Centricity Enterprise Web, version 3.0, GE Medical systems, FL, USA).

On the LLR, mechanical alignment was measured by measuring the difference between the femoral and tibial mechanical axes. Landmarks used were the center of the femoral head, the most proximal and central point of the femoral intercondylar notch, the mid-point of the tibial plateau, and the mid-point of the tibial plafond. Anatomical axes were measured in a similar fashion using the anatomical axis of the femoral and tibial shafts (Figure 3).

On the short-leg radiograph (SLR), anatomical axes were estimated using the intercondylar notch and the mid-point of the tibial plateau as central land marks as described above. The long axes of femoral and tibial shafts were then estimated by using the available length of the short-leg X-ray.

On both SLRs and LLRs, the alignment of femoral and tibial prostheses in the coronal plane were measured according to the system described by The Knee Society (12).

Reproducibility was performed on 25 subjects, extracted from the primary dataset using the random sample function in SPSS for Windows version 21.0 (SPSS Inc., Chicago, IL, USA), for all angles by two authors in order to determine inter- and intra-observer reliabilities. All reproducibility measures were conducted in a blinded fashion with no knowledge of prior results.

### Statistical analysis

Inter-rater reliability was assessed using the intra-class correlation coefficient (ICC) with a two-way mixed effects model and an absolute agreement definition (13). The one-sample *t*-test procedure was carried out to calculate the mean differences and SD between groups. The Bland–Altman method was also used to calculate the repeatability coefficient and limits of agreement (14). Statistical analyses were performed using SPSS for Windows version 21.0 (SPSS Inc., Chicago, IL, USA).

## Results

Radiographic analysis was performed on 75 subjects with a mean age of 69.7 years (range 50–88). Sixty percent of the cohort were female.

The mean mechanical axis on the LLR was 180.5°(SD 3.0, range 175.1–187.1), where values greater than 180° represent varus coronal plane alignment. Twenty-five knees in total had a mechanical axis more than 3° from neutral. Sixty-eight percent of these were in varus alignment. The mean offset between anatomical axis and mechanical axis as measured on the LLR was 6.4°(SD 0.8, range 4.8–8.7).

Inter-rater reliability analysis revealed “almost perfect” inter-observer and intra-observer reliabilities with values ≥0.81 on all measures on both SLRs and LLRs.

According to our hypothesis, we sought to determine if there was a correlation between the alignment measured on the LLR and that on the standard post-operative radiograph. Measurement of the mechanical axis on the LLR and anatomical axis on the SLR showed “substantial” agreement with values of 0.726 (*p* = 0.001) and 0.752 (*p* < 0.001), respectively. We compared mechanical axis values from the LLR with the anatomical axis measured on the SLR + 6°, which was the mean offset between mechanical and anatomical axes measured on the LLR. This calculation showed only “fair” agreement with an interclass correlation coefficient of 0.588 (*p* < 0.001).

There was less agreement between component alignment when comparing measurements from SLR and LLRs. Interclass correlation coefficient for tibial component alignment was 0.609 (*p* < 0.001) and for femoral alignment was 0.677 (*p* < 0.001). The correlation coefficient between anatomical axis measurements was 0.612 (*p* < 0.001).

**Figure 1:**
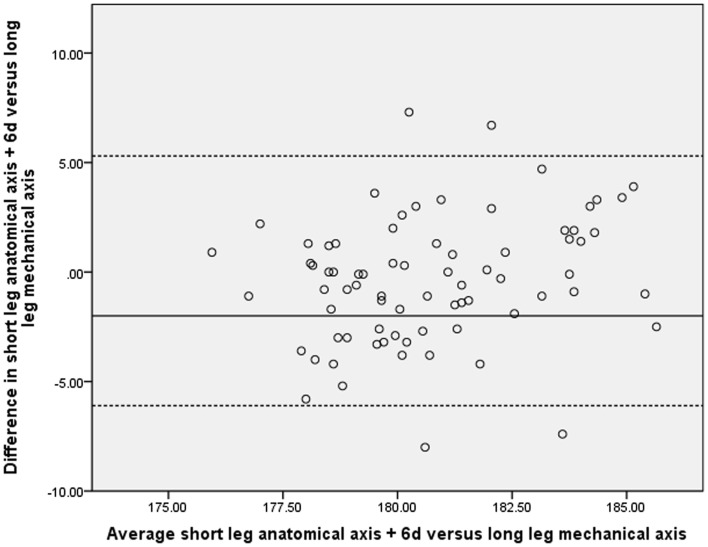
**Comparison of long and short-leg X-rays using Bland–Altman method**.

**Figure 2:**
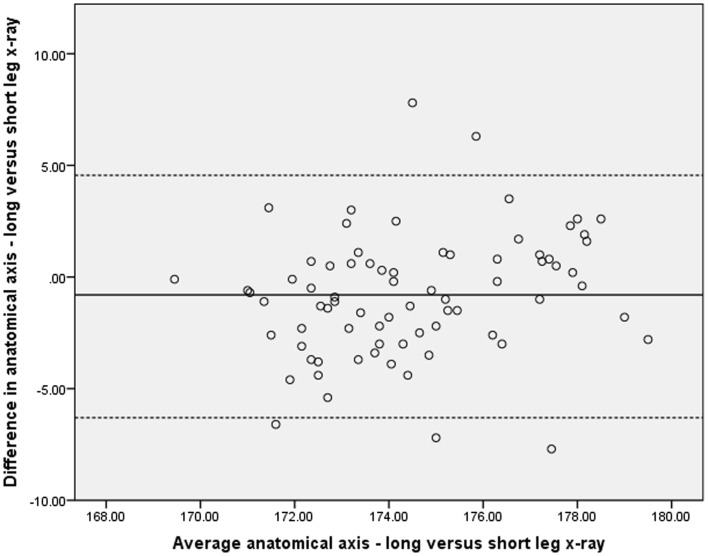
**Comparison of long and short-leg X-rays using Bland–Altman method**.

Given the statistical level of correlation between the above measures, it could be concluded that the in most cases the SLR could be used as a surrogate for the LLR. It is more important, however, to determine whether the variations between the two X-ray methods would lead to any clinical significance. As reported elsewhere correlation does not necessarily equate to agreement and as such the Bland–Altman method was used to assess the level of agreement between measures of alignment on both the SLRs and LLRs (15). These results are represented graphically (Figures 1 and 2) and demonstrate a significant level of disagreement that includes clinically important discrepancies of 5 or more degrees in 9% of cases.

**Figure 3:**
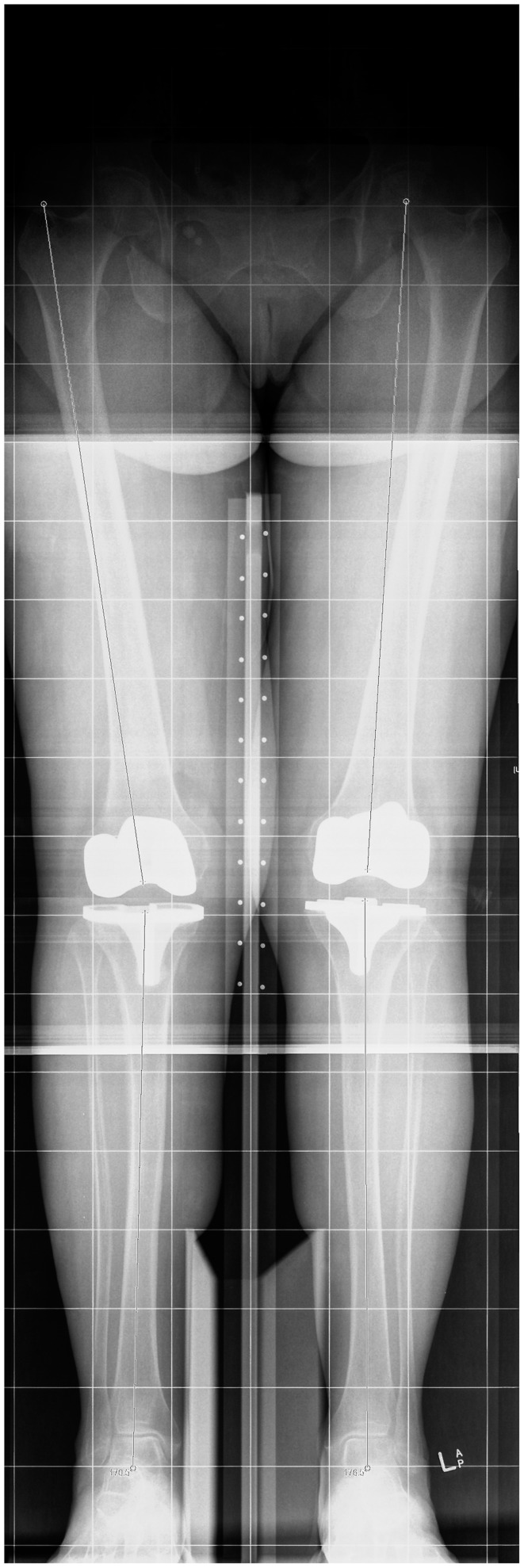
**Measurement of mechanical and anatomical axes on the long-leg X-ray**.

Using tibial component alignment on the short-leg X-ray may provide a surrogate marker for overall coronal plane alignment. Of the knees with a tibial component alignment less than 3° from neutral, as measured on the SLR, 98% had a mechanical axis within 3° of neutral. Conversely, of the 25 knees with a mechanical axis outside of 3° from neutral, only 28% had a tibial component alignment more than 3° from neutral on the short-leg X-ray.

## Discussion

Restoration of the lower limb mechanical axis to neutral is a primary aim of TKR surgery. Multiple studies have demonstrated the importance of a neutral coronal plane alignment in TKR correlating this with implant longevity, patient satisfaction, and overall function (1–4). The weight bearing LLR, using the hip, knee, and ankle as landmarks, is the gold standard investigation to measure coronal plane alignment. Despite this many surgeons, outside of a research setting, use only SLRs to assess alignment post TKR. Most often, the initial X-ray is taken in hospital on day one or two post-operatively with the patient in a supine position. This radiograph can be difficult to interpret due to variation in rotation and knee flexion, and in a supine position, may not adequately reflect the position of the knee in stance.

We hypothesized that the initial short-leg X-ray is an unreliable method of determining alignment following TKR surgery. Using the Bland–Altman method (Figure 1), we have shown that there is a significant lack of agreement between measures of alignment taken on the SLRs and LLRs. Significant variations of up to 7° were seen in a several cases.

A number of reasons are likely to play a role in this. Firstly, on the LLR, the anatomical axis of the leg is able to be calculated using the full length of the femoral and tibial shafts. On the short-leg X-ray, however, the plate is centered on the knee and only 10–20 cm of each of the femur and tibia can be used to estimate the anatomical axis. We have tried to mitigate this by using a 35 cm × 43 cm rather than a standard 24 cm × 30 cm cassette to enable more of the limb to be visualized. Despite this, the poor agreement between the anatomical axes on the two X-rays can be seen in Figure 2.

Secondly, the mechanical axis cannot be assessed on a standard knee X-ray as it requires visualization of both the hip and ankle. Previous studies have used the offset between anatomical and mechanical axes to allow estimation of the mechanical axis on a short-leg film. We calculated the mean offset on the LLR to be 6.4° (SD 0.8, range 4.8–8.7). Figure 1 shows a Bland–Altman plot of the anatomical axis plus an offset of 6° on the short-leg X-ray compared to the true mechanical axis measured on the LLR. Again, a high level of disagreement is seen. We also performed this calculation using varying offset values and found 6° to have the greatest level of agreement (Table 1).

**Table 1:** **Correlations – short- vs. long-leg X-rays**.

Measure	Intra-class correlation coefficient		Bland–Altman method for limits of agreement		
	ICC (95% CI)	*P*	Mean diff. (SD)	Repeatability coefficient	Limits of agreement
Tibia	0.609 (0.344, 0.762)	<0.001	0.8 (1.8)	3.5	−2.7, 4.3
Femur	0.677 (0.490, 0.796)	<0.001	0.5 (2.1)	4.1	−3.6, 4.6
FS-TS angle	0.612 (0.386, 0.754)	<0.001	−0.8 (2.8)	5.4	−6.3, 4.6
MA vs. SL-FS-TS	0.233 (−0.160, 0.556)	<0.001	5.6 (2.9)	5.7	−0.1, 11.3
MA vs. SL-FS-TS + 4 degrees	0.526 (0.197, 0.714)	<0.001	1.6 (2.9)	5.7	−4.1, 7.3
MA vs. SL-FS-TS + 5 degrees	0.583 (0.344, 0.735)	<0.001	0.6 (2.9)	5.7	−5.1, 6.3
MA vs. SL-FS-TS + 6 degrees	0.588 (0.350, 0.739)	<0.001	−0.4 (2.9)	5.7	−6.1, 5.3

Thirdly, and most importantly, the short-leg X-rays are taken in a supine position while the LLR is taken standing. The weight bearing X-ray represents the true functional position of the knee where both soft tissue and bony/prosthesis factors contribute to the overall alignment. Skytta et al. compared the LRR to a weight bearing short-leg X-ray of the knee following TKR and found excellent correlation between measures of alignment (16). While some limitations exist in Skytta’s statistical analysis, the difference between their results and those presented here may be explained by the short-leg X-ray being taken erect vs. supine.

Our results demonstrate good inter- and intra-observer reliabilities in the measures of alignment taken using a digital PACS system without the requirement for any additional software packages. In their study, McDaniel et al. found that using the tibial spines as the knee center was the most reliable method of assessing coronal alignment (10). In the replaced knee, we did not have the ability to use the spines as a landmark, and thus, opted to use a two-point system and the PACS Cobb angle tool. The highest point of the intercondylar notch was used as the femoral point and is a reliable and reproducible measure. Finding an appropriate tibial point is more difficult and in this study, we have used the mid-point of the tibial plateau. Most tibial prosthesis have a keel placed centrally in the coronal plane, and/or a locking mechanism for the polyethylene insert, and this can be used as an easy reference for the mid-point of the prosthesis; however, we found any tibial rotation could make identification of the mid-point difficult. Despite this, both inter- and intra-observer reliabilities were found to be excellent indicating that the measurement of alignment itself is unlikely to be a source of great error.

We sought to identify if using component position could be used as a marker for overall alignment. In particular, we found that using tibial alignment on the short-leg X-ray can provide a useful approximation of the mechanical axis. Of the patients with a mechanical axis of 180 ± 3° on the LLR, 98% of them had a tibial prosthesis placed within 3° of 90° to the long axis of the available tibial shaft on the short-leg X-ray. Only one patient who had a tibial cut greater than 3° from the perpendicular (total number of 8) had an overall mechanical axis within 3° of neutral. It may be that the only reliable method of estimating the alignment on the short-leg X-ray is to use the tibial component position as a surrogate marker.

Our paper has a number of weaknesses that must be acknowledged. Great efforts are undertaken to ensure standardization of X-ray technique but particularly when taking the post-operative X-rays some variability inevitably exists due to differences in the position of the limb that could have significant implication on measures of alignment. Measurements were undertaken using a digital PACS system with carefully determined landmarks but human error may play a role – a specific program to more accurately identify landmarks may have been useful. While consecutive cases have been assessed the retrospective nature of the radiological review involves inherent bias.

In conclusion, it is vital that surgeons performing total knee joint replacement surgery are able to accurately assess their ability to restore neutral coronal plane alignment post-operatively. In this study, we have shown that the current standard post-operative supine X-ray is an unreliable method of measuring alignment. We suggest that all surgeons should consider the routine use of the LLR in the post-operative assessment of total knee patients.

## Conflict of Interest Statement

The authors declare that the research was conducted in the absence of any commercial or financial relationships that could be construed as a potential conflict of interest.
